# PCAT19: the role in cancer pathogenesis and beyond

**DOI:** 10.3389/fcell.2024.1435717

**Published:** 2024-12-18

**Authors:** Haijun Hu, Hongliang Luo, Ziqing Deng

**Affiliations:** ^1^ Department of Anesthesiology, The Second Affiliated Hospital, Jiangxi Medical College, Nanchang University, Nanchang, Jiangxi, China; ^2^ Department of Gastrointestinal Surgery, The Second Affiliated Hospital, Jiangxi Medical College, Nanchang University, Nanchang, Jiangxi, China; ^3^ Department of General Surgery, Nanchang Third Hospital, Nanchang, Jiangxi, China

**Keywords:** long non-coding RNA, PCAT19, tumors, neuropathic pain, disease marker, therapeutic target

## Abstract

PCAT19, a long non-coding RNA, has attracted considerable attention due to its diverse roles in various malignancies. This work compiles current research on PCAT19’s involvement in cancer pathogenesis and progression. Abnormal expression of PCAT19 has been observed in various cancers, and its correlation with clinical features and prognosis positions it as a promising prognostic biomarker. Additionally, its ability to effectively differentiate between tumor and normal tissues suggests significant diagnostic value. PCAT19 exhibits a dual nature, functioning either as an oncogene or a tumor suppressor, depending on the cancer type. It is implicated in a range of tumor-related activities, including cell proliferation, apoptosis, invasion, migration, metabolism, as well as tumor growth and metastasis. PCAT19 acts as a competing endogenous RNA (ceRNA) or interacts with proteins to regulate critical cancer-related pathways, such as MELK signaling, p53 signaling, and cell cycle pathways. Furthermore, emerging evidence suggests that PCAT19 plays a role in the modulation of neuropathic pain, adding complexity to its functional repertoire. By exploring the molecular mechanisms and pathways associated with PCAT19, we aim to provide a comprehensive understanding of its multifaceted roles in human health and disease, highlighting its potential as a therapeutic target for cancer and pain management.

## 1 Introduction

Long non-coding RNAs (lncRNAs) are RNA molecules typically longer than 200 nucleotides and lack protein-coding potential (Yarmishyn and Kurochkin, 2015; Mattick et al., 2023). Recently, lncRNAs have garnered significant attention and emerged as crucial regulators in a broad range of biological processes and diseases (Lin Y. et al., 2023; Xue et al., 2023; Nemeth et al., 2024; Liu et al., 2024; Zhang et al., 2024; Wang et al., 2024). They participate in regulating alternative splicing, cell differentiation, cell cycle progression, and gene expression at various levels (e.g., chromatin modification, transcription, and post-transcriptional processing) (Boo and Kim, 2020; Ferrer and Dimitrova, 2024; Herman et al., 2022; Sebastian-delaCruz et al., 2021; He et al., 2019; Pisignano and Ladomery, 2021; Statello et al., 2021). Increasing evidence identifies lncRNAs as oncogenes or tumor suppressors, playing pivotal roles in tumor development (Gao et al., 2020; Liao et al., 2024; Huarte, 2015; Bach and Lee, 2018; Zhao et al., 2022).

The Homo sapiens (human) Prostate Cancer Associated Transcript 19 (PCAT19), also known as Long Intergenic Non-Protein Coding RNA 1190 (LINC01190), classified as a lncRNA, is situated on chromosome 19q13.2. This gene consists of four exons and spans 46,481 nucleotides. PCAT19 has been identified as a significant player in human pathological disease (Oo et al., 2022; Wang Z. et al., 2022; Liu et al., 2022), including both oncological (Gao et al., 2018; Hua et al., 2018; Xu et al., 2019; Zhang X. et al., 2019; Xie and Hu, 2020) and neurological contexts (Huo et al., 2022; Zhao et al., 2024). PCAT19 was initially characterized in prostate cancer (PCa) (Hazelett et al., 2014). It has been associated with tumor progression and poor prognosis in PCa (Gao et al., 2018; Hua et al., 2018). Further studies have expanded its relevance to other cancer types, including laryngocarcinoma (Xu et al., 2019), lung cancer (Zhang X. et al., 2019), and glioma (Xie and Hu, 2020), where it demonstrates abnormal expression and plays multifaceted roles in tumorigenesis. PCAT19 shows significant clinical value, impacting clinical features, prognosis, and diagnosis across multiple human cancers (Xu et al., 2019; Zhang X. et al., 2019; Feng et al., 2023; Acha-Sagredo et al., 2020; Xiao et al., 2022; Masoud and Mohamadynejad, 2024). It is implicated in regulating cell proliferation, migration, invasion, and apoptosis through various molecular mechanisms (Xu et al., 2019; Zhang X. et al., 2019; Xie and Hu, 2020; Xiao et al., 2022; Tang et al., 2021), such as interacting with miRNAs and modulating key signaling pathways. These findings underscore PCAT19’s potential as a promising cancer biomarker and an attractive target for therapeutic intervention.

In addition to its oncological significance, recent research has unveiled the involvement of PCAT19 in neuropathic pain (Huo et al., 2022; Zhao et al., 2024), a chronic pain condition resulting from nerve injury. Neuropathic pain presents a substantial clinical challenge due to its complex pathophysiology and limited treatment options. The role of lncRNAs in neuropathic pain is an emerging field (Li et al., 2019; Wu et al., 2019), and PCAT19 has been highlighted for its contribution to the molecular mechanisms underlying pain sensitization and maintenance (Huo et al., 2022; Zhao et al., 2024). Studies have shown that PCAT19 regulates neuropathic pain via the miR-182-5p/JMJD1A (Huo et al., 2022) and miR-378a-3p/KDM3A axis (Zhao et al., 2024). These interactions are implicated in facilitating microglia activation and neuroinflammation, thereby accelerating chronic neuropathic pain (Huo et al., 2022; Zhao et al., 2024).

This work aims to provide a comprehensive overview of the current understanding of PCAT19’s role in tumors and neuropathic pain. We discuss the mechanisms by which PCAT19 influences cancer progression and its potential as a therapeutic target. Additionally, we explore the emerging evidence of PCAT19’s function in neuropathic pain, shedding light on its dual role in these two distinct yet critically important health conditions. By elucidating the roles of PCAT19, this review seeks to highlight its potential for clinical applications in both oncology and pain management.

## 2 PCAT19 expression in human tumors

### 2.1 PCAT19 expression in cancer specimens

Recent studies have detected dysregulated expression of PCAT19 across various human cancer specimens (Xu et al., 2019; Zhang X. et al., 2019; Xie and Hu, 2020; Feng et al., 2023; Acha-Sagredo et al., 2020; Xiao et al., 2022; Tang et al., 2021; Wang B. et al., 2022). Notably, PCAT19 is upregulated in several cancers, including glioma, laryngocarcinoma, gastric cancer (GC), and PCa (Table 1). This upregulation suggests that PCAT19 may play a oncogenic role in the progression or maintenance of these cancer types. Conversely, PCAT19 is significantly downregulated in breast cancer, lung cancer, colorectal cancer (CRC), and endometrial cancer (Table 1). This downregulation indicates a different role of PCAT19 in these cancers, possibly acting as a tumor suppressor.

**TABLE 1:** Expression of PCAT19, its prognostic value, and diagnostic relevance in various cancer types.

Cancer type	Expression	Significant clinical variables	End-points	Unfavorable	ROC analysis	References
Glioma	Upregulated	—	—	—	—	Xie and Hu (2020)
Laryngocarcinoma	Upregulated	Tumor stage	Overall survival	High expression	—	Xu et al. (2019)
Breast cancer	Downregulated	Axillary lymph node, pathological grade, clinical stage, T stage, regional lymph node	Overall survival, disease-free survival	Low expression	—	Feng et al. (2023)
Non-small cell lung cancer	Upregulated	—	Overall survival	High expression	—	Zhang et al. (2019a)
Non-small cell lung carcinoma	Downregulated	Tumour stage, tumour histology, tumor depth	—	—	AUC = 0.990 (tumour vs. normal tissue)	Acha-Sagredo et al. (2020)
Lung adenocarcinoma	Downregulated	T; N; M; age, tumor stage	Overall survival	Low expression	—	Tang et al. (2021)
Lung cancer	Downregulated	Tumor size; Pathology	Overall survival	Low expression	—	Wang et al. (2022b)
Gastric cancer	Upregulated	Tumor size, lymphatic metastasis, TNM stage	Overall survival, Disease-free survival	High expression	—	Xiao et al. (2022)
Colorectal cancer	Downregulated	Metastatic status	—	—	AUC = 0.940 (tumour vs. normal tissue)	Masoud and Mohamadynejad (2024)
Prostate cancer	Upregulated	—	Biochemical recurrence-free	High expression (patients carrying rs11672691 GG)	—	Gao et al. (2018)
Prostate cancer	—	—	Disease-free survival	High expression	—	Hua et al. (2018)
Bladder cancer	Upregulated	Tumor size, stage, grade, lymph node metastasis, distant metastasis	Overall survival	High expression	—	Wang and Jiang (2022)
Endometrial cancer	Downregulated	Tumour grade, tumour stage	Overall survival	Low expression	—	Wang et al. (2021b)

**FIGURE 1:**
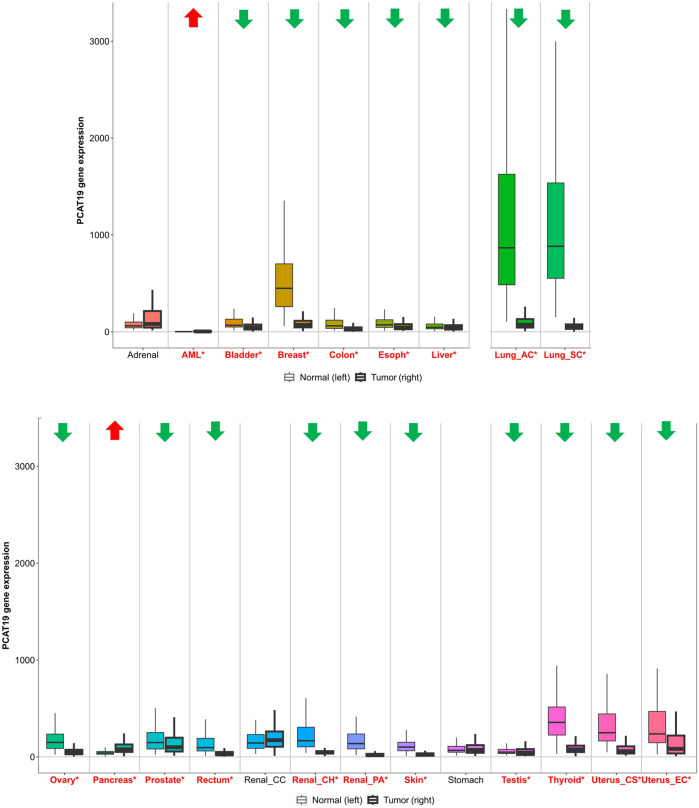
PCAT19 expression levels in tumors vs. normal tissues. Cancer types with significantly upregulated PCAT19 are marked in red with upward arrows; those with downregulated PCAT19 are in green with downward arrows. Significant differences are indicated by red asterisks.

Furthermore, a comprehensive analysis using TNMplot (https://tnmplot.com/analysis/) provided an in-depth perspective on PCAT19 expression across diverse cancer types (Figure 1). TNMplot is a specialized bioinformatics tool designed to visualize and analyze gene expression patterns across various cancer types, leveraging multiple databases to offer insights into tumor biology (Bartha and Győrffy, 2021). Here, significant upregulation of PCAT19 was observed in acute myeloid leukemia (AML) and pancreatic cancer, reinforcing the potential oncogenic role of PCAT19 in these malignancies. On the other hand, obvious downregulation was noted in most tumors, including those from the bladder, breast, colon, esophagus, liver, lung, ovary, prostate, rectum, kidney, skin, testis, thyroid, and uterus. Notably, there is inconsistency between the results from TNMplot and the findings from the literature reports, such as in prostate cancer, which may indeed arise from several factors, including differences in sample size, methodology, or the characteristics of the patient cohorts analyzed. Overall, the widespread abnormal expression of PCAT19 across various cancer types suggests a crucial role for PCAT19 in tumorigenesis and development.

### 2.2 PCAT19 expression in cancer cell lines

**FIGURE 2:**
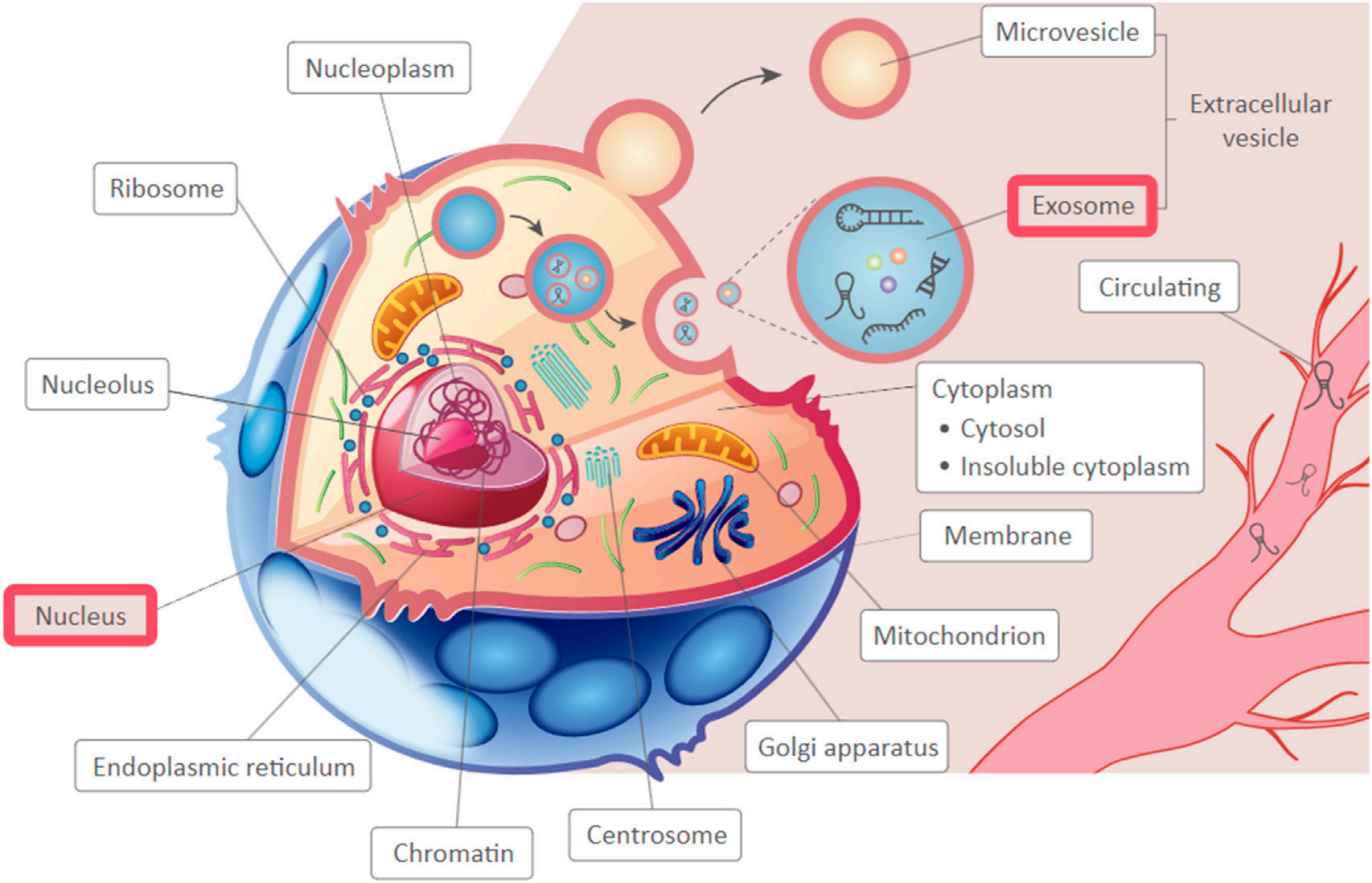
The predicted sub-cellular localization of PCAT19, highlighting identified locations in a red frame.

The expression of PCAT19 was also investigated in several cancer cell lines (Xie and Hu, 2020; Feng et al., 2023; Acha-Sagredo et al., 2020; Xiao et al., 2022; Tang et al., 2021; Wang B. et al., 2022). It has been showed dysregulated expression, with upregulation in glioma (Xie and Hu, 2020) and GC (Xiao et al., 2022) cell lines compared to normal cell lines, and downregulation in breast cancer (Feng et al., 2023), lung cancer (Acha-Sagredo et al., 2020; Tang et al., 2021; Wang B. et al., 2022) cell lines. This consistent pattern of dysregulation in both tissue specimens and cell lines underscores the potential importance of PCAT19 in cancer biology. Interestingly, PCAT19 lncRNA was predominantly localized in the cytoplasm of various cancer cell types, including lung cancer cells (A549, SK-MES-1) (Wang B. et al., 2022) and GC cells (AGS, MGC-803) (Xiao et al., 2022), and highly enriched in the nuclear fraction of PCa cells (LNCaP, V16A) (Hua et al., 2018). The cytoplasmic localization of PCAT19 indicates it might interact with other cytoplasmic molecules, potentially affecting processes like mRNA stability, translation, or signaling pathways. Additionally, as a nuclear lncRNA, PCAT19 could influence target gene expression by interacting with protein complexes. Additionally, based on the predicted subcellular localization of PCAT19 using LnCeCell (http://bio-bigdata.hrbmu.edu.cn/LnCeCell/) (Wang P. et al., 2021), a database that provides insights into lncRNA-associated gene networks within a single cell, PCAT19 is suggested to localize in the nucleus and exosomes, as illustrated in Figure 2. This multi-compartmental presence indicates that PCAT19 may serve diverse functions within the cell, contributing to its complex role in cancer pathology.

## 3 Clinical values of PCAT19 in human cancers

PCAT19 has emerged as a crucial biomarker n various human cancers due to its significant dysregulation and its association with key clinicopathological parameters, prognosis, and diagnostic utility, as summarized in Table 1. Overall, the dysregulation of PCAT19 across multiple cancer types, its correlation with key clinicopathological parameters, and its prognostic and diagnostic significance underscore its clinical value. PCAT19 not only serves as a biomarker for tumor prognosis and diagnosis but also offers potential as a therapeutic target, making it a focal point in cancer research and treatment strategies.

### 3.1 Clinical features and prognostic value of PCAT19 in human cancers

Across different cancer types, PCAT19 exhibits a consistent pattern of altered expression, strongly correlated with tumor progression and patient outcomes. For instance, in laryngeal cancer (Xu et al., 2019), PCAT19 is markedly overexpressed in tumor tissues compared to adjacent normal tissues, with higher levels observed in advanced stages (III and IV). This overexpression is linked to poorer overall survival (OS), underscoring its potential role in tumor aggressiveness and patient prognosis. In GC (Xiao et al., 2022), higher PCAT19 expression significantly correlates with larger tumor size, lymphatic metastasis, and advanced TNM stage. GC patients with high PCAT19 expression present a poorer OS rate than those with low PCAT19 expression. In PCa, PCAT19 is significantly upregulated in tumor samples (Gao et al., 2018). Although PCAT19 does not predict biochemical recurrence-free survival in general, PCa patients with the rs11672691 GG genotype and higher PCAT19 expression are at increased risk of biochemical relapse (Gao et al., 2018). Additionally, higher PCAT19-long expression is associated with poorer disease-free survival (DFS) in PCa patients from the TCGA cohort (Hua et al., 2018). In bladder cancer (Wang and Jiang, 2022), patients with high PCAT19 expression have significantly lower survival rates and worse prognosis, with notable differences in tumor size, T staging, tumor grade, lymph node metastasis, and distant metastasis.

In contrast, in breast cancer (Feng et al., 2023), PCAT19 expression is notably downregulated. This lower expression is associated with favorable clinical features, such as lower tumor grade, earlier clinical stage, and less lymph node metastasis. This inverse relationship suggests that PCAT19 may act as a protective factor in breast cancer, where its downregulation predicts better OS and DFS (Feng et al., 2023). Similarly, in CRC, PCAT19 is downregulated in both metastatic and non-metastatic tumor tissues compared to normal tissues (Masoud and Mohamadynejad, 2024). In lung cancer, Zhang et al. (Zhang X. et al., 2019) revealed that PCAT19 is upregulated in non-small cell lung cancer (NSCLC), with higher levels associated with shorter OS compared to cases with lower levels of PCAT19. However, subsequent studies (Acha-Sagredo et al., 2020; Tang et al., 2021; Wang B. et al., 2022)have shown that PCAT19 is significantly downregulated in lung cancer, including NSCLC and lung adenocarcinoma (LUAD). Lower levels of PCAT19 are associated with advanced clinical pathological features, including T, N, and M stages, and poorer OS outcomes. In endometrial cancer (Wang Z. et al., 2021), PCAT19 is significantly downregulated in cancer tissues, with its expression negatively associated with tumor grade and stage, and lower PCAT19 expression is indicative of shorter OS in endometrial cancer.

In addition, several PCAT19-containing prognostic models, such as a TIME-related signature in stomach adenocarcinoma (Zhu et al., 2022), necroptosis-related lncRNA signatures in bladder cancer (Jin et al., 2023), and necroptosis-related lncRNA signatures in uterine corpora endometrial cancer (Lin Z. et al., 2023), have been developed and demonstrated significant prognostic value in these cancers.

### 3.2 Diagnostic potential of PCAT19 in human cancers

The diagnostic potential of PCAT19 is significant, characterized by its differential expression between tumor and normal tissues, rendering it a valuable biomarker for cancer diagnosis. For example, in lung cancer (Acha-Sagredo et al., 2020), PCAT19 exhibits high expression in NSCLC tissues compared to adjacent normal tissues, demonstrating strong discriminatory power with a high area under the curve. Similarly, in CRC (Masoud and Mohamadynejad, 2024), ROC analysis shows that PCAT19 effectively distinguishes between tumor and normal tissues with high sensitivity and specificity (AUC = 0.940), highlighting its clinical utility in CRC diagnostics.

## 4 Impacts and mechanisms of lncRNA PCAT19 on human malignancies

Research has investigated the role of PCAT19 in various human tumors using both cell lines and mouse models (Table 2). PCAT19 has been implicated in the pathogenesis and progression of multiple cancers, including glioma (Xie and Hu, 2020), laryngocarcinoma (Xu et al., 2019), breast cancer (Feng et al., 2023), lung cancer (Zhang X. et al., 2019; Acha-Sagredo et al., 2020; Tang et al., 2021; Wang B. et al., 2022), GC (Xiao et al., 2022), PCa (Gao et al., 2018; Hua et al., 2018), and bladder cancer (Wang and Jiang, 2022). Interestingly, PCAT19 demonstrates a dual nature, acting as both an oncogene and a tumor suppressor depending on the cancer type. This dual functionality highlights the complex regulatory networks in which PCAT19 is involved. For instance, in glioma (Xie and Hu, 2020), laryngocarcinoma (Xu et al., 2019), and GC (Xiao et al., 2022), PCAT19 functions as an oncogene, promoting cell proliferation, migration, and invasion by interacting with various miRNAs and signaling pathways. Conversely, in breast cancer (Feng et al., 2023) and LUAD (Tang et al., 2021), PCAT19 exhibits tumor-suppressive properties, inhibiting these cellular processes.

**TABLE 2:** Diverse roles of PCAT19 in different human tumors: *in vivo, in vitro*, and molecular mechanisms.

Cancer type	*In vitro* experiment cell lines	Cell expression	Significant cellular functions	Animal models	Significant experiment phenotypes	Related molecule/ pathway	Role	References
Glioma	U87, U251, LN-229 and HA cell lines	Upregulated	Cell proliferation, invasion, and apoptosis	Xenograft tumor model	Tumor growth (tumor weight)	PCAT19/miR-142-5p/MELK signaling	Tumorigenic	Xie and Hu (2020)
Laryngocarcinoma	HEp‐2 and AMCHN‐8 cell lines	—	Cell proliferation and metabolism; cell‐cycle transition	Xenograft tumor model	Tumor growth (tumor volume and weight)	PCAT19/ miR‐182/PDK4 axis	Tumorigenic	Xu et al. (2019)
Breast cancer	SKBR3, T47D, MCF7, MDA, MB231, HS578T, and MCF10A cell lines	Down-regulated	Cell proliferation	Xenograft tumor model	Tumor growth (tumor size)	—	Anticancer	Feng et al. (2023)
Non-small cell lung cancer	H1993 cell line	—	Cell proliferation	—	—	p53	Tumorigenic	Zhang et al. (2019a)
Non-small cell lung carcinoma	A549, Calu-1, Calu-3, Calu-6, COR-L23, DMS53, H358, H2073, HTB-59, HTB-182, LUDLU-1, SK-LU-1, SKMES-1 and IMR-90 cell lines	Down-regulated	—	—	—	—	—	Acha-Sagredo et al. (2020)
Lung adenocarcinoma	A549, SPC-A1, and 16HBE cell lines	Down-regulated	Cell proliferation, migration, and invasion	—	—	PCAT19/miR-143-3p/mRNA regulatory network	Anticancer	Tang et al. (2021)
Lung cancer	A549, H1299, SK-MES-1, and BEAS-2B cell lines	Down-regulated	Cell proliferation, apoptosis, and cycle	Tumor-bearing model	Tumor growth (tumor volume and weight)	PCAT19/miR-25-3p/MAP2K4 signaling axis	Anticancer	Wang et al. (2022b)
Gastric cancer	AGS, MGC-803, MKN45, SGC-7901, BGC-823 and GES1 cell lines	Up-regulated	Cell viability, proliferation, invasion, and apoptosis	—	—	SP1/PCAT19/miR-429/DHX9	Tumorigenic	Xiao et al. (2022)
Prostate cancer	22Rv1, Du145, LNCaP cell lines	—	Cell proliferation, migration and invasion	—	—	rs11672691/HOXA2/PCAT19 and CEACAM21	Tumorigenic	Gao et al. (2018)
Prostate cancer	LNCaP, V16A and 22Rv1 cells	—	Cell proliferation, migration and invasion	Xenograft tumor model	Tumor growth (tumor volume and weight) and metastasis	PCAT19-long/HNRNPAB/cell-cycle genes	Tumorigenic	Hua et al. (2018)
Bladder cancer	T24 cells	—	Cell vitality, proliferation, migration, invasion and apoptosis	Tumor-bearing model	Tumor growth (tumor size)	PCAT19/miR-335-5p/IER2	Tumorigenic	Wang and Jiang (2022)

Mechanistically, PCAT19 plays a crucial role in tumor progression by acting as a competitive endogenous RNA (ceRNA). CeRNAs are RNA molecules that can bind to shared microRNAs (miRNAs), effectively sequestering these miRNAs and preventing them from degrading their target mRNAs. This interaction alleviates the miRNA-induced degradation of target mRNAs, thereby stabilizing them. CeRNAs represent a prevalent mechanism for regulating post-transcriptional gene expression, influencing both physiological processes and pathological conditions (Yang et al., 2021; Qi et al., 2015; Salmena et al., 2011; Li et al., 2022; Tang et al., 2024). Several studies have demonstrated that PCAT19 plays a crucial role in tumorigenesis by sequestering specific microRNAs. Notably, it interacts with miR-142-5p (Xie and Hu, 2020), miR-182 (Xu et al., 2019), miR-143-3p (Tang et al., 2021), miR-429 (Xiao et al., 2022), and miR-335-5p (Wang and Jiang, 2022), thereby modulating the expression of target genes. Additionally, PCAT19 interacts directly with key proteins. For instance, the transcription of PCAT19 is influenced by allele G of rs11672691, which enhances the chromatin binding affinity of HOXA2 and affects PCa cellular properties (Gao et al., 2018). Furthermore, PCAT19-long interacts with HNRNPAB to activate a subset of cell-cycle genes associated with PCa progression (Hua et al., 2018), thereby promoting tumor growth and progression. Its involvement also extends to critical signaling pathways, including MELK (Xie and Hu, 2020), MAP2K4 (Wang B. et al., 2022), and p53 (Zhang X. et al., 2019) signaling pathways, underscoring its intricate role in cancers. The following section details the functional roles of PCAT19 in different type of tumors.

## 5 Functions and regulatory roles of PCAT19 in different tumors

### 5.1 Glioma

Gliomas, the most common primary brain tumors, are characterized by significant heterogeneity and resistance to conventional therapies (Ou et al., 2020; Rong et al., 2022; Finch et al., 2021; Miller et al., 2021). Recent research has highlighted the critical role of the PCAT19 in glioma progression (Xie and Hu, 2020). PCAT19 is significantly upregulated in glioma tissues, where it acts as a molecular sponge for miR-142-5p, which has been reported to facilitate cell communication and can affect cancer cell behaviors in multiple tumors (Zareifar et al., 2024; Yu et al., 2019). The interaction between PCAT19 and miR-142-5p leads to the upregulation of MELK, which has been found to enhance tumor cell proliferation, invasion, and resistance to apoptosis (Tang et al., 2020; Ganguly et al., 2015; Thangaraj et al., 2020). *In vitro* experiments have demonstrated that knockdown of PCAT19 or overexpression of miR-142-5p decreases glioma cell proliferation, colony formation, and invasion, while promoting apoptosis by modulating the expression of Cyclin B1, CDK2, N-cadherin, Bcl-2, Bax, and E-cadherin. Additionally, *in vivo* studies using glioma xenograft models have shown that silencing PCAT19 significantly reduces tumor growth. These findings suggest that targeting the PCAT19/miR-142-5p/MELK axis could serve as a novel therapeutic strategy for glioma, providing new avenues for effective treatment interventions.

### 5.2 Laryngeal cancer

Laryngocarcinoma, a common type of head and neck cancer, has complex underlying mechanisms that are not fully understood (Argiris et al., 2008; Johnson et al., 2020). Recent studies have highlighted the critical role of PCAT19 in the progression of this cancer (Xu et al., 2019). *In vitro* experiments using laryngeal cancer cell lines HEp-2 and AMC-HN-8 demonstrated that knocking down PCAT19 reduces cell proliferation, enhances mitochondrial respiration, and inhibits glycolysis. Specifically, the knockdown decreased the expression of Pyruvate Dehydrogenase Kinase 4 (PDK4) and reduced phosphorylation of Pyruvate Dehydrogenase E1 Alpha (PDHE1α), key regulators of cellular metabolism (Chen et al., 2024; Thoudam et al., 2022; Yonashiro et al., 2018; Zhang J. et al., 2019). Moreover, miR-182 plays a crucial role in tumorigenesis, exerting significant influence in diverse facets of malignancy such as proliferation and metastasis (Ma et al., 2022; Sameti et al., 2023a; Stafford and McKenna, 2023). Here, miR-182 was identified as a mediator between PCAT19 and PDK4, impacting cellular metabolism and proliferation. The PCAT19/miR-182/PDK4 axis regulates cell proliferation by modulating glycolysis and mitochondrial respiration, promoting the Warburg effect—a metabolic hallmark of cancer (Liberti and Locasale, 2016; Schwartz et al., 2017; Chen X. et al., 2015). *In vivo* xenograft tumor models further confirmed that PCAT19 knockdown significantly reduces tumor growth, increases miR-182 levels, and decreases PDK4 expression and PDHE1α phosphorylation. These findings suggest that targeting the PCAT19/miR-182/PDK4 axis could be a promising therapeutic strategy for laryngeal cancer, offering new avenues for effective treatment interventions.

### 5.3 Breast cancer

Breast cancer is the most prevalent malignancy affecting women worldwide, posing significant challenges due to its high incidence and potential for metastasis (Wilkinson and Gathani, 2022; Harbeck et al., 2019). Recent studies have highlighted the involvement of lncRNAs in breast cancer progression (Sideris et al., 2022; Jin et al., 2021; Fonseca-Montaño et al., 2023; Jiang et al., 2023), with PCAT19 emerging as a significant tumor suppressor (Feng et al., 2023). Bioinformatic analyses and machine learning models identified PCAT19 as crucial for prognosis, with high expression levels associated with lower clinical stages and reduced lymph node metastasis. *In situ* hybridization (ISH) assays confirmed that PCAT19 expression is lower in breast cancer tissues compared to normal tissues. Functional assays demonstrated that knocking down PCAT19 in breast cancer cell lines MCF-7 and T47D promoted cell proliferation, while overexpression inhibited tumor growth *in vivo*. Specifically, knockdown increased cell proliferation and showed a trend towards enhanced migration and invasion, though not statistically significant. Conversely, overexpressing PCAT19 reduced tumor size in mouse xenograft models. These findings underscore the tumor-suppressive role of PCAT19 in breast cancer, suggesting its potential as a prognostic biomarker and therapeutic target, providing new insights for risk stratification and treatment strategies for BC patients.

### 5.4 Lung cancer

Lung cancer, particularly NSCLC, which includes LUAD, remains the leading cause of cancer-related deaths worldwide (Bray et al., 2024; Araghi et al., 2023; Duma et al., 2019). PCAT19 has been studied extensively for its dual roles in lung cancer (Zhang X. et al., 2019; Tang et al., 2021; Wang B. et al., 2022). In NSCLC, specifically using the H1993 cell line, PCAT19 has been identified as an oncogene (Zhang X. et al., 2019). Research shows that PCAT19 is upregulated in NSCLC tissues and correlates with poor patient survival. This oncogenic role is linked to the negative regulation of the tumor suppressor gene p53 (Zhang X. et al., 2019). Silencing PCAT19 in H1993 NSCLC cells increases p53 expression and decreases cell proliferation, while overexpression of PCAT19 reduces p53 levels and enhances proliferation. These findings suggest that PCAT19 promotes NSCLC progression by inhibiting the p53 tumor-suppressive pathway, underscoring a critical mechanism through which PCAT19 facilitates NSCLC development.

In contrast, studies involving other NSCLC cell lines (such as A549, SK-MES-1 and SPC-A1) indicate a tumor-suppressive role for PCAT19 (Tang et al., 2021; Wang B. et al., 2022). In these studies, PCAT19 expression is downregulated in LUAD tissues and cell lines, with higher expression levels associated with better prognosis (Tang et al., 2021; Wang B. et al., 2022). Functionally, PCAT19 inhibits LUAD cell proliferation, migration, and invasion (Tang et al., 2021). The regulatory mechanism involves the PCAT19/miR-143-3p axis, where PCAT19 acts as a ceRNA for miR-143-3p. MiR-143-3p is known to regulate target genes involved in tumor biology through various signaling pathways across multiple types of tumors (Wu et al., 2024; Asghariazar et al., 2023). In LUAD, the PCAT19/miR-143-3p axis modulates MAPK signaling pathway, thereby influencing cellular behaviors (Tang et al., 2021). In addition, PCAT19 regulates the proliferation and apoptosis of lung cancer cells by inhibiting miR-25-3p via targeting the MAP2K4 signal axis (Wang B. et al., 2022). These contrasting roles highlight the influence of cell line context on PCAT19 function, emphasizing the importance of considering cellular context and specific genetic backgrounds in PCAT19 research. Overall, PCAT19 demonstrates context-dependent roles in lung cancer, acting as an oncogene in NSCLC by inhibiting p53 and as a tumor suppressor in LUAD by acting as ceRNA dynamics. This dual functionality underscores PCAT19’s potential as both a prognostic biomarker and a therapeutic target in lung cancer, providing new avenues for tailored treatment strategies.

### 5.5 Gastric cancer

Gastric cancer is a prevalent and lethal malignancy, particularly common in East Asia (Shin et al., 2023; Rahman et al., 2014; Baccili Cury Megid et al., 2023), necessitating a deep understanding of its molecular mechanisms for better diagnosis and treatment. The PCAT19 has emerged as a significant player in GC progression (Xiao et al., 2022). Bioinformatics and experimental analyses have shown that PCAT19 is upregulated in GC tissues, correlating with advanced clinical features, and poorer prognosis (Xiao et al., 2022). Mechanistically, PCAT19 acts as an oncogene in GC, driven by the transcription factor SP1, which enhances PCAT19 transcription by binding to its promoter region (Xiao et al., 2022). In the cytoplasm, PCAT19 functions as ceRNA by sponging miR-429, a prominent tumor suppressor miRNA implicated in various cancers, including GC (Guo et al., 2020; Sheng et al., 2018; Zhang et al., 2016). By binding to miR-429, PCAT19 impedes its ability to downregulate DHX9, an RNA helicase (Gulliver et al., 2020; Leone et al., 2017). This sponging action leads to increased DHX9 expression, promoting GC cell proliferation and invasion (Xiao et al., 2022). Experimental knockdown of PCAT19 in GC cell lines (such as AGS and MGC-803) via siRNA significantly suppresses cell proliferation and invasion while increasing apoptosis, as evidenced by assays such as CCK-8, EdU, and flow cytometry (Xiao et al., 2022). These findings underscore PCAT19’s role as an oncogene in GC, highlighting its potential as a therapeutic target and prognostic biomarker.

### 5.6 Prostate cancer

Prostate cancer (PCa) remains a prevalent and deadly disease among men, particularly in the Western world (Rawla, 2019; Zhang et al., 2023; Bergengren et al., 2022). Research has revealed that genetic factors play a significant role in PCa, with heritability estimates reaching 57% (Pagadala et al., 2022; Mucci et al., 2016). Among these genetic factors, single nucleotide polymorphisms (SNPs) have been extensively studied, revealing several loci associated with PCa susceptibility (Zhang et al., 2019c; Turner et al., 2012; Zhang P. et al., 2018). One such SNP, 19q13 rs11672691, within the intronic region of a lncRNA gene, PCAT19, has been linked specifically to aggressive forms of PCa, suggesting a crucial role in disease progression and prognosis (Amin Al Olama et al., 2013; Shui et al., 2014). And studies have validated this association to PCAT19 and defined a biological mechanism of PCAT19 in PCa (Gao et al., 2018; Hua et al., 2018; Xia and Wei, 2018). Gao et al. (2018) validated this association and defined an elegant biological mechanism underlying the 19q13 locus, therefore likely informing aggressive PCa poor prognosis and treatment. Functional analyses have shown that the G allele of rs11672691 is significantly correlated with higher expression levels of PCAT19 and CEACAM21 (Gao et al., 2018). These genes, previously unlinked to PCa, now appear to play significant roles in its pathology. Knockdown experiments of PCAT19 in PCa cell lines demonstrated that reduced expression of PCAT19 leads to decreased cell proliferation, migration, and invasion (Gao et al., 2018). Conversely, overexpression of PCAT19 promotes these malignant behaviors, indicating that PCAT19 enhances the aggressiveness of PCa cells (Gao et al., 2018). Further investigations into the regulatory mechanisms of PCAT19 expression revealed that the rs11672691 region acts as an active enhancer, marked by specific epigenetic modifications and binding of several transcription factors, including the androgen receptor (AR), HOXB13, ERG, and the newly identified HOXA2. Notably, HOXA2, which binds more strongly to the G allele of rs11672691, is an androgen-responsive gene essential for PCa cell growth and invasiveness. This suggests that HOXA2, in conjunction with the rs11672691 enhancer, regulates PCAT19 expression. PCAT19 itself exhibits enhancer-like functions, particularly in regulating the expression of CEACAM21. Chromatin conformation capture assays (3C-qPCR) revealed a direct chromatin loop between the PCAT19 and CEACAM21 loci, indicating a physical interaction that facilitates their coordinated regulation. This long-range chromatin interaction underscores the complexity of genetic regulation in PCa. In addition, Hua et al. (Hua et al., 2018) further demonstrated the functional mechanisms of rs11672691 in PCa progression through the upregulation of a lncRNA isoform, PCAT19-long. And PCAT19-long interacts with HNRNPAB and drives the progression of PCa through the upregulation of a subset of cell-cycle genes associated with PCa progression, thereby promoting PCa tumour growth and metastasis (Hua et al., 2018). Collectively, PCAT19 plays a crucial role in the progression and aggressiveness of PCa. Its regulation by genetic variants and interaction with other oncogenic factors like HOXA2 and HNRNPAB highlights its importance in PCa biology and its potential as a target for therapeutic intervention.

### 5.7 Bladder cancer

Bladder cancer is a common urological malignancy with a complex pathogenesis involving numerous genetic and molecular alterations (Lenis et al., 2020a; Lenis et al., 2020b). Recent research has identified the PCAT19 as a crucial regulator in the malignant progression of bladder cancer through the miR-335-5p/IER2 axis (Wang and Jiang, 2022). PCAT19 is upregulated in bladder cancer tissues and is negatively correlated with miR-335-5p expression. miR-335-5p has been reported to be dysregulated in tumors and acts as a potential player in tumor initiation, development, and metastasis (Ye et al., 2021; Sandoval-Bórquez et al., 2017; Zhang et al., 2019d; Liu et al., 2019). Mechanistically, PCAT19 acts as a ceRNA by sponging miR-335-5p, thereby reducing its availability and leading to the upregulation of IER2, a downstream target gene (Wang and Jiang, 2022). This regulatory interaction enhances bladder cancer cell proliferation, migration, and invasion while inhibiting apoptosis. Functional assays, including MTT, flow cytometry, and transwell assays, demonstrated that PCAT19 knockdown significantly reduces these oncogenic behaviors, whereas overexpression of PCAT19 promotes them (Wang and Jiang, 2022). *In vivo* studies using a mouse xenograft model corroborated these findings, showing that PCAT19 knockdown leads to smaller tumor volumes and reduced expression of proteins associated with cell survival and invasion, such as Survivin, Bcl-2, and MMP-9, while increasing pro-apoptotic Bax levels (Wang and Jiang, 2022). Conversely, overexpression of PCAT19 resulted in larger tumors and the opposite protein expression patterns (Wang and Jiang, 2022). These results underscore the pivotal role of the PCAT19/miR-335-5p/IER2 signaling axis in bladder cancer progression, highlighting PCAT19’s potential as a therapeutic target.

## 6 Effects of lncRNA PCAT19 on neuropathic pain

LncRNAs are a class of regulatory RNA molecules that do not encode proteins but play crucial roles in gene expression regulation (Wang and Chang, 2011). They constitute approximately 40% of the RNA expressed specifically in the brain (Zimmer-Bensch, 2019; Derrien et al., 2012). Recent studies indicate that lncRNA expression responds to neuronal activity and injury, influencing nervous system development and synaptic plasticity by regulating neuronal outgrowth, differentiation and synapse formation (Aprea et al., 2013; Belgard et al., 2011; Mercer et al., 2008; Wu et al., 2010; Barry et al., 2014; Lipovich et al., 2012). Importantly, lncRNAs play pivotal roles in the development of neuropathic pain by modulating ion channels and neuroinflammation (Li et al., 2019; Wu et al., 2019), both of which are central to its pathogenesis (Vicario et al., 2020; Luo and Huang, 2024).

Among these, lncRNA PCAT19 has emerged as a key player in neuropathic pain (Huo et al., 2022; Zhao et al., 2024). One study revealed that PCAT19 regulates neuropathic pain through the miR-182-5p/JMJD1A pathway (Huo et al., 2022). miR-182-5p, a well-researched microRNA, is recognized not only for its significant role in tumorigenesis (Sameti et al., 2023b; Souza et al., 2022; Zhou et al., 2023) but also as a crucial inflammatory mediator in immune-related diseases (Wan et al., 2019; Blaya et al., 2016). Similarly, JMJD1A acts as a vital regulator in cancer development (Yang et al., 2022; Manni et al., 2022; Fan et al., 2023) and is implicated in processes involving inflammation and oxidative stress (Zhao et al., 2021; Qu et al., 2023). In the miR-182-5p/JMJD1A pathway, PCAT19 acts as a sponge for miR-182-5p, preventing it from downregulating JMJD1A. By maintaining higher levels of JMJD1A, PCAT19 influences gene expression patterns that contribute to the persistence of neuropathic pain. Another study (Zhao et al., 2024) delved into the molecular interactions involving PCAT19 in neuropathic pain. This study also focused on the ceRNA mechanism, where PCAT19 acts as a decoy for miR-378a-3p. By sequestering miR-378a-3p, PCAT19 prevents it from inhibiting KDM3A, an lysine demethylase (Arifuzzaman et al., 2020; Zhang Q. J. et al., 2018). The upregulation of KDM3A leads to the demethylation and subsequent activation of BDNF genes, which are crucial for pain signaling and microglia activation (Smith, 2024; Huang et al., 2021; Thakkar and Acevedo, 2023). This pathway highlights the complex interplay between lncRNAs and miRNAs in regulating neuroinflammatory processes that drive chronic neuropathic pain.

## 7 Future perspectives

PCAT19, an important regulatory lncRNA, exhibits clinical relevance and plays a multifaceted role in various human cancers. PCAT19 has the potential to serve as a prognostic and diagnostic biomarker, as detailed in Table 1. Specifically, in laryngeal carcinoma (Xu et al., 2019), breast cancer (Feng et al., 2023), lung cancer (Zhang X. et al., 2019; Tang et al., 2021; Wang B. et al., 2022), GC (Xiao et al., 2022), bladder cancer (Wang and Jiang, 2022), and endometrial cancer (Wang Z. et al., 2021), the expression level of PCAT19 is significantly associated with OS. Moreover, in breast (Feng et al., 2023), gastric (Xiao et al., 2022), and prostate cancers (Hua et al., 2018), the expression of PCAT19 is significantly linked to DFS. Additionally, PCAT19 demonstrates diagnostic capability in lung cancer (Acha-Sagredo et al., 2020) and CRC (Masoud and Mohamadynejad, 2024), effectively distinguishing between tumor and normal tissues. Notably, the functions of PCAT19 vary significantly depending on the tumor type, as summarized in Table 2. In several cancers, including glioma (Xie and Hu, 2020), laryngocarcinoma (Xu et al., 2019), GC (Xiao et al., 2022), PCa (Gao et al., 2018; Hua et al., 2018), and bladder cancer (Wang and Jiang, 2022), PCAT19 acts as an oncogene, modulating cellular processes such as proliferation, migration, invasion, and tumor growth. Conversely, in breast cancer (Feng et al., 2023) and LUAD (Tang et al., 2021; Wang B. et al., 2022), PCAT19 exhibits tumor-suppressive properties, where its overexpression inhibits cell proliferation, migration, invasion, and tumor growth. This dual role emphasizes the complex nature of PCAT19 in cancer development, functioning as either an oncogene or tumor suppressor based on the cellular environment and regulatory networks involved.

To further elucidate the mechanisms by which PCAT19 acts as either an oncogene or a tumor suppressor, it is essential to explore its molecular interactions across various cancer types. The differential expression of PCAT19 between normal and cancerous tissues suggests that its levels significantly impact crucial cellular pathways involved in cancer progression. When acting as an oncogenic lncRNA, PCAT19 activation leads to increased expression, resulting in unchecked cancer cell growth and tumor progression. For instance, in PCa (Gao et al., 2018; Hua et al., 2018), upregulation of PCAT19 regulates the cell cycle-associated pathways and promotes uncontrolled cell growth and metastasis, indicating its oncogenic role in this context. Therefore, combining PCAT19 inhibition with chemotherapy or immunotherapy could potentially enhance treatment outcomes for cancers where it functions as an oncogene. Conversely, when functioning as a tumor suppressor, the downregulation or inactivation of PCAT19 removes constraints on cancer cell proliferation, indicating its intricate regulatory potential in tumor biology. For example, in breast cancer (Feng et al., 2023), the downregulation of PCAT19 might result in the loss of its tumor-suppressive functions, leading to increased tumor proliferation and aggressiveness. Hence, enhancing PCAT19 expression through oligonucleotide therapies, in combination with chemotherapy or immunotherapy, could improve patient outcomes in cancers where it acts as a tumor suppressor.

In addition to its roles in cancer, recent studies have implicated PCAT19 in neuropathic pain (Huo et al., 2022; Zhao et al., 2024). PCAT19 regulates neuropathic pain through epigenetic mechanisms, particularly via the miR-182-5p/JMJD1A (Huo et al., 2022) and miR-378a-3p/KDM3A (Zhao et al., 2024) pathways, both of which influence pain sensitization and maintenance. Targeting the epigenetic pathways influenced by PCAT19 presents an innovative approach to treating chronic pain conditions. By focusing on PCAT19-related therapies in combination with existing treatments, there is potential to enhance therapeutic effectiveness. Modulating crucial molecular pathways involved in pain sensitization and maintenance through PCAT19 targeting could offer promising new avenues for pain relief.

The implications of these findings for clinical applications are substantial. Understanding PCAT19’s roles in both cancer and neuropathic pain can inform treatment strategies and enhance patient outcomes. In cancers where PCAT19 functions as an oncogene, strategies aimed at inhibiting its expression or disrupting its molecular interactions may prove to be effective. Conversely, in cancers where PCAT19 acts as a tumor suppressor, approaches to enhance its expression could provide significant therapeutic benefits. Furthermore, the exploration of PCAT19’s role in neuropathic pain management underscores the need for targeted interventions. Investigating potential therapeutic approaches to influence PCAT19 expression or its interactions with associated miRNAs could offer promising new strategies for addressing chronic pain.

The dual role of PCAT19 as both an oncogene and tumor suppressor, along with its involvement in neuropathic pain, underscores its potential as an important disease biomarker in clinical settings. Future *in vivo* and *in vitro* research should focus on elucidating the precise molecular mechanisms by which PCAT19 regulates gene expression and cellular processes across a broader range of cancer types. And since chromosome-specific aneuploidy is observed in approximately 90% of cancers (Dodgson et al., 2016; Weaver and Cleveland, 2006; Nagaoka et al., 2012; Chen G. et al., 2015), and this phenomenon can have profound implications for cancer biology, including the modulation of specific genes and regulatory pathways. When considering PCAT19, a lncRNA implicated in multiples tumors, it is important to investigate whether and how chromosomal abnormalities might influence its expression and function. Investigating the chromosomal status in tumor samples could help establish a correlation between aneuploidy and the expression levels of PCAT19, and the changes in chromatin structure and transcription factor binding in aneuploid cells may disrupt normal regulatory networks, thereby affecting the expression of PCAT19 and influencing cellular behaviors such as proliferation, apoptosis, and metastasis. Therefore, given that aneuploidy is prevalent in many cancers, exploring the impact of chromosome-specific aneuploidy on PCAT19 across different tumor types may reveal common mechanisms and further enhance our understanding of the relationship between genomic instability and cancer. Furthermore, large-scale clinical studies are also warranted to validate its utility in predicting patient outcomes, diagnostic value, and guiding treatment decisions, ultimately harnessing the therapeutic potential of PCAT19 in the management of cancer and chronic pain.

## 8 Conclusion

PCAT19 is a multifaceted lncRNA with significant roles in both cancer and neuropathic pain. It functions as either an oncogene or tumor suppressor in different cancers by acting as a ceRNA, interacting with key proteins, and regulating signaling pathways, thereby affecting a series of tumor activities. Additionally, emerging evidence suggests its important involvement in neuropathic pain modulation. Understanding the regulatory mechanisms and pathways of PCAT19 is crucial for developing targeted therapies for cancer and pain management. Future research should focus on these mechanisms to fully harness PCAT19’s therapeutic potential.
